# 

**Published:** 1881-03

**Authors:** 


PAGE.

ORIGINAL COMMUNICATIONS:

Statistics Relating to a Scarlatina Epidemic. By Joseph Fowler, M. D., . . . . 337

CLINICAL REPORTS:

Abscess of the Antrum of Highmore Simulating Acute Inflammation of the Lachrymal Sac. Reported by B. H. Grove, M. D., From the Practice of Dr. Lucien Howe, . 343

Prof. T. F. Rochester's Medical Clinic at the Buffalo General Hospital. Reported by Frederick Peterson, M. D., . . . . 346

Notes on Two Cases of Compound Comminuted Fracture of the Skull. By Dr. S. H. Benton, . . . . . . . . . 350

TRANSLATIONS:

A Case of Suppurative Pleuritis with Gangrene of a Portion of Lung Tissue and its Escape from the Operation Wound. From the German by P. W. Van Peyma, M. D., 352

SELECTIONS:

Travers vs. Boardman: An Action of Tort, and What It Teaches, . . . . . 356

Natural Bone-Setting, . . . . . . . 360

Statistics of Medical Education in the United States, . . . . . . . . . . 364

Therapeutic Review for 1880, . . . . 365

The Determination of the Sexes, . . . . 368

EDITORIAL:

Medical Department of the University of Buffalo, . . . . . . . . 369

Insane Asylum—the Alleged Abuses in their Administration, . . . . . . . 378

International Medical Congress, . . . . 381

Royal Infirmities, . . . . . . . . 382

REVIEWS, . . . . . . . 383

